# Effects of Supplementing Quails’ (*Coturnix japonica*) Diets with a Blend of Clove (*Syzygium aromaticum*) and Black Cumin (*Nigella sativa*) Oils on Growth Performance and Health Aspects

**DOI:** 10.3390/life12111915

**Published:** 2022-11-17

**Authors:** Kamlah Ali Majrashi

**Affiliations:** Biological Sciences Department, College of Science & Arts, King Abdulaziz University, Rabigh 21911, Saudi Arabia; kmajrashi@kau.edu.sa

**Keywords:** clove oil, *Nigella sativa* oil, Japanese quail, growth, carcass, blood components

## Abstract

In an attempt to discover a safe growth promoter and partial alternative for antibiotics, this existing study explores the efficacy of using assorted levels of cold-pressed oil mixtures consisting of 1:1 clove and black cumin (*Nigella sativa*) oils (CLNS) against the indices of growth and carcass traits, as well as blood components of growing Japanese quails. In a complete randomized design, three hundred growing unsexed Japanese quails (one week of age) were included in this experiment. The treated groups were as follows: (1) control basal diet (CLNS0), (2) basal diet + 1.50 mL CLNS/kg diet (CLNS1.5), and (3) basal diet + 3.00 mL CLNS/kg diet (CLNS3). The results showed that supplementing the diet with a 3.00 mL CLNS/kg diet insignificantly improved body weight (BW) compared with the CLNS0 and CLNS1.5 groups. A significantly (*p* < 0.05) higher feed intake and feed conversion ratio—FCR— (deterioration of feed conversion) were reported after the addition of CLNS. Feeding the quails on a 3.00 mL CLNS/kg enriched-diet yielded superior values of dressing percentage, carcass yield, and breast and thigh relative weights compared to other groups. A significant decline was noticed in creatinine and BUN levels in birds fed a 1.50 and 3.00 mL CLNS/kg diet compared with the CLNS0 group The liver enzymes and total bilirubin activities showed insignificant effects in quails fed CLNS-enriched diets. The total protein and globulins concentrations presented a significant augment in quails that received CLNS. The antiradical activity of CLNS supplementation showed increases in hepatic reduced glutathione (GSH) activity and the levels of superoxide dismutase (SOD), glutathione peroxidase (GPx), catalase, glutathione S transferase (GST), and glutathione reductase (GR) in birds. The concentration of MDA in hepatic homogenates that received CLNS-diets was significantly decreased compared with the control quails. These findings clarified that the dietary inclusion of CLNS can enhance the growth performance and antioxidative status of growing Japanese quails.

## 1. Introduction

Medicinal plants, i.e., basil (*Ocimum basilicum*), thyme (*Thymus vulgaris*), sage (*Salvia officinalis*), clove (*Syzygium aromaticum*), and *Nigella sativa* have been applied in different food supplements, representing a digestive equilibrium of gut microbiota and a functional part connected to growing precise enzyme synthesis with substantial antimicrobial action [1,2,3]. Herbal cold-pressed oils are a significant source of valuable nutritionally essential fatty acids, bioactive molecules, soluble lipids, phenols, natural antioxidants, vitamins, and minerals [3,4]. Phenols exhibited nutritional significance with natural antimicrobial and antioxidants properties. They might directly diminish the synthesis of oxidative stress (OS) and avoid lipid peroxidation (LP), as well could improve health statuses and prevent certain diseases [5,6]. The usage of herbs, their bioactive constituents, or cold-pressed oils presently has beneficial effects on the livestock industry as well as health care schemes because of their comprehensive positive effects, such as enhancing growth efficiency, immune improvement, and maintenance of reproductive health [5,7]. Many investigations clarified that birds’ immune systems were diminished through the imbalance between high OS production and decreased antioxidant capacity in the cellular system [8]. Thereafter, the machinery of oxidation by OS in the living system has gained significant attention through utilizing natural components [9]. Moreover, reactive oxygen species (ROS) are formed through the body’s regular system through using oxygen molecules such as with respiration and phagocytosis [9].

In the era of free antibiotics in the livestock industry, scientists have been focusing their studies on combating ROS synthesis and improving the release of antioxidants factors for enhancing animal productivity [1,4,7]. Previous studies on poultry demonstrated that the dietary inclusion of black seed or clove oils [2,10] as natural products had enormous potential for enhancing health and immunity profiles [11]. They have various pharmacological properties, such as antioxidant and anti-inflammatory, because of their richness with phenolic compounds [12,13]. Additionally, it has been revealed that this improvement may be attributed to the antioxidant capacity of those oils, oils which have been used individually or with other essential oils [12,14]. However, the blend of clove oil and black seed oil (CLNS) has not been explored in quail diets for enhancing growth and health status. Based on the antioxidant effect [12,13] of dietary cold-pressed oils such as black seed and clove oils, it was hypothesized that the dietary supplementation of this blend (CLNS) in the diet of growing Japanese quail could enhance growth indices, blood biochemistry, and carcass traits, as well as the health status. Therefore, the objectives of this work were to study the impacts of cold-pressed oil mixtures (CLNS) on growth efficiency, carcass quality, and blood components of growing Japanese quails. A further study investigating the economic efficiency of using such additives is recommended.

## 2. Materials and Methods

### 2.1. Diets, Birds, and Experimental Procedure

For this experiment, 300 one-week-old growing Japanese quails 23.76 ± 0.13 g in initial body weight were included in a completely randomized design trial with three treated groups. Birds were randomly allotted into three treated categories of 5 replicates, 20 unsexed birds per replicate. The mixture consisting of 1:1 clove and black cumin (*Nigella sativa*) oils (CLNS) was added in three doses (0, 1.50, and 3.00 mL/kg diet). The treatments were as follows: (1) Control basal diet (CLNS0), (2) Basal diet + 1.50 mL CLNS /kg diet (CLNS1.5), and (3) Basal diet + 3.00 mL CLNS/kg diet (CLNS3). The experimental period lasted five weeks, and during this period, birds were given the basal diet with or without oil to fulfill the birds’ requirements [15]. Table 1 depicts the constituents and chemical components of the basal diet employed for growing quails. The birds were housed in the same environmental conditions, where the temperature was kept at 24 °C for 24 h of light (the same type, intensity, color of light) during the experimental period. The animals were kept in a traditional cage (50 × 30 × 50 cm^3^; 1500 cm^2^ of floor space) with feed and fresh water offered ad libitum.

### 2.2. Data Collection

#### 2.2.1. Growth Performance

For assessment of the growth performance, birds were weighed individually via an electrical balance (the accuracy = 0.01 g) at weekly intervals to get the BW (body weight) and BWG (body weight gain), whereas the values of FI (feed intake) and FCR (feed conversion ratio; g feed/g gain) were detected on a weekly basis for determining the feed efficiency. During the whole experimental time, the value of morality in growing quails was naught. At the end of treating the animals, six birds (each group) were randomly chosen to assess the carcass traits at six weeks of age. Animals were manually sacrificed as recommended by the Islamic method. The hot carcasses were weighed, and the weights of the heart, thigh, abdominal fat, liver, gizzard, and breast were documented and expressed as g/kg of slaughter weight according to our prior work [7]. The dressed and carcass weights were studied (dressed weight = carcass weight plus giblets weight/live body weight). Giblets included the heart, liver, and gizzard.

#### 2.2.2. Biochemical Analysis

To assess the blood biochemical in birds after five weeks of treatment, birds were fasting overnight. Then, the blood samples were gathered via the scarifying of six birds (each group) into non-heparinized tubes. Then, the samples were exposed to centrifugation at 2795 g for 15 min at 4 °C to acquire the serum. After that, it was reserved at −20 °C pending biochemical examination. Liver function in the blood serum of treated and control groups, including the serum values of AST (aspartate aminotransferase), ALT (alanine aminotransferase), and total bilirubin were assessed following the commercial kits’ instructions and by colorimetric methods. The serum of the total protein fraction and albumin were evaluated by the colorimetric technique according to the manufacturer’s guidelines of commercially offered kits (Biodiagnostic Company, Giza, Egypt). However, total globulins were considered by subtracting albumin values from total protein values. To assess the kidney function, BUN (blood urea nitrogen) and creatinine levels in the serum of quails were estimated based on the commercially available kit. Additionally, for serum lipid profile examination, FFA (free fatty acids; [16]), total cholesterol [17], LDL-c (Low-density lipoprotein-cholesterol), HDL-c (high-density lipoprotein-cholesterol; [18]), and triacylglycerol [19] were determined using commercially available kits following the manufacturer’s instructions.

#### 2.2.3. Hepatic Antioxidant Indices

Hepatic antioxidant and oxidative markers were evaluated as earlier defined in our prior description [20]. Concisely, one gram of liver tissues was weighed, sterilized and homogenized via chilled potassium chloride (1.17%), and then the samples were centrifuged at 2795 g at 4 °C. After complete centrifuging, the supernatant was picked up, put into sterilized Eppendorf tubes, and kept at −20 °C for further examinations. The hepatic activities of lipid peroxidation (MDA; malondialdehyde), GPx (glutathione peroxidase), GR (glutathione reductase), GSH (reduced glutathione), CAT (catalase), SOD (superoxide dismutase), and GST (glutathione S transferase) were detected [21] using commercial kits (Biodiagnostics, Giza, Egypt) and following the instructions.

### 2.3. Statistical Analysis

All data presented in this experiment were analyzed by ANOVA analysis using a completely randomized design using the GLM procedures of SAS [22]. In addition, the differences between means were tested via post-hoc Tukey’s test. The statistical significance was considered at *p <* 0.05

## 3. Results

### 3.1. Growth Performance

The data in Table 2 provide details about the influence of the dietary presence of CLNS on the growth indices of growing Japanese quails. It is obvious that the impacts of CLNS on the values of BW were significant (*p <* 0.05) only at six weeks of age. Supplementing the diet with 3.00 mL CLNS/kg diet increased BW compared to the CLNS0 and CLNS1.5 groups. A similar trend was observed for BWG. Results showed that the best (*p <* 0.007) BWG was given in the CLNS3 group, which exceeded the control values at 3–6 and 1–6 weeks of age, respectively, compared with the CLNS0 group. The data in Table 3 reveal that FI was statistically (*p* < 0.01) different due to CLNS inclusion in the first weeks of the experiment and the entire period (1–3 weeks of age). Moreover, feed intake increased as the level of CLNS increased. However, FCR was insignificantly affected by dietary treatments.

### 3.2. Carcass Traits

Consequences existing in Table 4 show the impacts of CLNS dietary inclusion on the carcass features of fatting quails. Results revealed that all carcass traits, except giblets’ relative weight, were impacted (*p <* 0.01) due to CLNS supplementation. Feeding the quails on a 3.00 mL CLNS/kg enriched-diet yielded superior values of the dressing, carcass, and breast and thigh relative weights compared to other groups.

### 3.3. Blood Biochemical Parameters

The safety of CLNS supplementation for Japanese quails is indicated in Table 5. The serum AST, ALT, and total bilirubin activities exhibited insignificant effects on quails fed CLNS-enriched diets compared with CLNS0 treatment. It is worth observing that CLNS insignificantly decreased liver enzyme activity and albumin. However, the total protein and total globulins concentrations significantly improved in both treatments, CLNS1.5 and CLNS3, compared with the CLNS0 group. There were non-statistically significant fluctuations detected in albumin concentrations among the experimental groups and a significant decline in creatinine and BUN levels in birds fed a 1.50 and 3.00 mL CLNS/kg diet compared with the CLNS0 group (Table 6).

The hypolipidemic and anti-atherogenic possibilities of CLNS are shown in Table 6 and are indicated by a substantial depression in total cholesterol, triacylglycerol, BUN, creatinine, LDL-c, and FFA levels in growing Japanese quails that received the 1.50 and 3.00 mL CLNS/kg diet compared with the control. Nevertheless, the level of HDL-c revealed an increase (*p <* 0.05) in birds that consumed CLNS-enriched diets compared to the control. The antiradical activity of CLNS supplementation showed an improvement in hepatic GSH, CAT, SOD, GPx, catalase, GR, and GST activities in growing quails (Table 7). Conversely, the concentration of MDA in hepatic homogenates that received CLNS-diets significantly decreased compared with the control quails.

## 4. Discussion

Judging by the existing experiment, the use of an essential oils blend including clove and black cumin (*Nigella sativa*) oil supplementation (at different levels of 1.5 or 3 mL/kg diet) had a significant influence on the body weight gains (especially in CLNS3 group) feed intake, total protein and globulins concentrations, dressing, carcass, breast and thigh relative weights of growing Japanese quails. As well as the antiradical activity of CLNS, supplementation showed a significant improvement of hepatic GSH, SOD, GPx, catalase, GR, and GST activities in quails. On the other hand, the gained information suggested that the addition of CLNS to the quails’ diets had no effect on feed efficiency, liver enzymes, and total bilirubin activities. It was telling that CLNS addition at level 3 ml/kg diet was the most effective dose in enhancing quails’ productive performance and antioxidant capability compared with 1.5 mL of CLNS.

In the last three decades, there have been a huge bulk of scientific reports regarding the use of natural compounds of essential oils in enhancing the growth, productivity, and reproductive efficiency of farm animals, including poultry [10,14,20,23], because of their safety, effectiveness, and applicable and easy manufacturing, as well as their remarkable anti-microbial, antioxidant, and anti-inflammation qualities [6,13]. Several studies illustrated the positive properties of essential oils in improving the productivity and growth of poultry by enhancing the digestibility and absorption of nutrients so that they could be used as growth promoters [11,23,24,25]. Similar to our obtained data, Ertas et al. [26] suggested that the addition of blended essential oils (anise, clove, and oregano oils) in chick diets enhanced body weight gain (BWG).

Feeding chicks with clove oil (600 mg/kg diet) significantly improved final BW and BWG. They suggested that these improvements in growth indices may be due to its high content of some bioactive agents, such as eugenol, eugenyl acetate, β-cariofileno, α-humulene, and β-caryophyllene [13], which could boost the feed digestibility and nutrients absorption via stimulating the secretions of digestive enzymes [7,11]. Adding 2% black seed powder to the diet of growing quails significantly enhanced the growth performance including BWG, FBW, and feed efficiency [2]. It was mentioned that the Nigellone molecule (60–80%) is the main compound in the essential oil of black seeds [12]. This molecule was found to have antioxidant and antimicrobial properties that enhance growth performance [26]. Improving feed palatability due to CLNS addition may be the reason for increasing FI. Furthermore, Abd El-Hack et al. [27] stated that improving FI may enhance palatability when enriching a diet with black seed oil. In a previous study, [28] pointed out that broiler chicks fed the diet added with 600 mg/kg clove oil presented increased FI compared to the basal diet group. Moreover, feeding growing quails with a 1.5 mL/kg diet improved the BWG and FI [10]. In addition, an earlier study on chicks [26] evidenced the constructive influence of clove oil on intestinal enzymes and feed utilization with high doses of clove essential oil. This improvement could be attributed to clove oil being super rich in manganese, zinc, and other trace elements required for carbohydrate and protein metabolism and the synthesis of cholesterol and fatty acids. A previous study demonstrated that *Nigella sativa* can directly or indirectly stimulate thyroid hormone secretions by activating pituitary gland secretions [29]. Avci et al. [30] discovered that stimulating the synthesis of thyroid hormones is critical for body metabolism, and improving carbohydrates and amino acid consumption via the fastening of their metabolism. On the contrary, [31] assumed that dietary black cumin seed extract did not significantly impact the FI in growing quails.

For carcass traits, our obtained data are in accordance with those confirmed by Isable and Santos [32], who clarified that percentages of breast and carcass were greater (*p <* 0.001) in chicks fed a blend essential oil of cinnamon and clove oils (100 mg/kg diet) than those in other treated groups. Several previous experiments agree with our findings in the data obtained by [2,23]. In our previous work, adding clove oil enhanced the carcass traits of Japanese quails compared with basal diets [27].

The data obtained from the current research were inconsistent with the previous data on chicks [33]. They found no clove oil or powder effect on the BW or BWG. Those authors suggested that the high levels of clove oil inclusion may have produced inadequate levels to affect growth. The same effect was detected in the broiler, as reported previously by [34,35]. Moreover, Azaegan et al. [36] did not detect any significant enhancement in the percentage of the carcass in the broiler given 450 mg/kg of clove oil after 42 days of treatment. Recently, black seed powder administration (1 or 2 or 4%) had no notable influence on the carcass features of growing quails [2]. The use of essential oil blends in quail diets needs further experiments to determine the potential synergistic effect of two or more essential oils.

The effect of CLNS addition on liver function assures the beneficial impact of black cumin oil in improving the hepatic functions and, after that, the welfare and health status of the birds [37]. The present data are similar to the previous data reported by Al-Beitawi and Ghousein [38], who found an enhancement in broiler liver function after feeding black cumin.

The main active component of clove is eugenol, with 90–95% oil content, and which is an effective polyphenolic molecule [13]. Eugenol has several pharmacological applications, including having antidiabetic, antioxidant, anti-hyperlipidemic, anti-inflammatory, and hypolipidemic effects [12,39].

Studies have reported that eugenol substantially reduces serum cholesterol levels by constraining the lipogenesis process in hepatic tissues, therefore strongly signifying that eugenol may protect against fatty liver diseases in different animal studies [10,14,40]. As a support to our verdicts, the anti-hyperlipidemic role of eugenol was detected by lowering the concentration of LDL-c, triacylglycerol, and total cholesterol compared with lovastatin, a lipid-lowering drug [41]. Additionally, Hussien et al. [10] showed a significant reduction in triacylglycerol, total cholesterol, LDL, and free fatty acids in quails fed diets added with 0.75–1.5 mL of clove oil. The hypolipidemic action of clove oil may be associated with its richness of eugenol compound. In this sense, the molecular mechanisms regarding the hypolipidemic activity of clove oil needed further explorations that are not considered in the current study.

During intensive poultry production, birds are exposed to several stressor issues (stock density, temperature, light, etc.) that diminish the body’s antioxidant defense. Thereafter, providing a suitable immunomodulator mediator, especially in natural products, could maintain homeostasis in the body of birds [8]. Oxidative stress occurs when ROS synthesis exceeds the antioxidant system’s capability to guard the cellular system contra-oxidized compounds. In the current work, the blended oils of clove and black seed (3 mL/kg diet) significantly enhanced the antioxidant capability of quails by augmenting GSH, CAT, SOD, GR, GPx, and GST activities as well as reducing lipid peroxidation (MDA). The antioxidant action of clove oil may be linked to eugenol, which forms an iron-oxygen chelate complex with the help of an allyl group in its structure and preserves copper and iron in the reduced form [12].

Clove oil supplementation can prevent the initiation of hydroxyl radicals, which represent the secondary products of lipid peroxidation. Similar to our data, some researchers found that the addition of clove oils to the diets of quails induced an increase in the levels of serum antioxidant enzyme activities (CAT, SOD, GPx and GST), as well as significantly depressed the levels of MDA, protein carbonyl (as an indicator of protein oxidation), and DNA damage markers (8-hydroxy-2′-deoxyguanosine) [10,11]. The previous results reflect the potentiality of using clove oils as a good antioxidant agent [7,13] and modulating lipid oxidation, and after that, protecting cell organelles from damage and supporting health status. Eugenol can also vigorously function as a tool for decreasing LDL, safeguarding hepatic tissues from severe steatosis, lowering inflammation stressors, and enhancing antioxidant profiles [41].

Recently, quails [2] or broiler [23] diets supplemented with black seed oils improved antioxidant status and reduced lipid peroxidation, thus promoting birds’ productivity and growth performance. This improvement was attributed to its ability to enhance antioxidant defense, decrease protein and lipid peroxidation, and contribute to DNA impairment [9], and which thereafter evidence the birds’ defenses alongside oxidative stress that affected the overall growth and productivity performance of the birds [33]. The present study revealed that the blend of clove and black seed oils enhanced growth and feed efficiency, carcass traits, blood biochemicals, antioxidant capacity, and reduced the LP. It seems that the blend of essential oils could be a growth promoter for a sustainable health status in quails.

## 5. Conclusions

The present study revealed that the supplementation of CLNS in growing Japanese quails’ diets enhanced growth performance, carcass characteristics, liver function, and other health aspects. CLNS showed excellent antiradical activity. It improved the activity of hepatic GSH and the levels of SOD, GPx, catalase, GST, and GR. In addition, the concentration of MDA in hepatic homogenates significantly decreased in CLNS-treated birds compared with the control. Therefore, the use of CLNS is recommended as a safe growth promoter. Regarding the inclusion level, the 3.00 mL CLNS/kg diet displayed the best effect on growth performance and health parameters, but higher inclusion levels of CLNS merits investigation by including economic considerations.

## Figures and Tables

**Table 1:** Composition and chemical analysis of the basal diet.

Ingredients	%
Gluten meal	3.20
Soybean meal	38.69
Yellow Corn	53.03
Soybean oil	1.67
Di Calcium phosphate	0.81
Vit-min Premix *	0.30
NaCl	0.11
Limestone	0.30
DL Methionine	0.39
L-Lysine HCl	1.50
Calculated analysis **:	
Crude protein, %	24.04
Metabilozable energy, Kcal/kg	2903
Calcium, %	0.85
Available phosphorous, %	0.45
Methionine + Cysteine, %	0.88
Lysine, %	1.60

* Growth Vitamin and Mineral premix. Each 2 kg consists of Vit A 12,000,000 IU; Vit D3, 2000,000 IU; Vit. E. 10 g; Vit k3 2 g; Vit B1, 1000 mg; Vit B2, 49 g; Vit B6, 105 g; Vit B12, 10 mg; Pantothenic acid, 10 g; Niacin, 20 g, Folic acid, 1000 mg; Biotin, 50 g; Choline Chloride, 500 mg, Fe, 30 g; Mn, 40 g; Cu, 3 g; Co, 200 mg; Si, 100 mg, and Zn, 45 g. ** Calculated according to NRC [15].

**Table 2:** Body weight and body weight gain of Japanese quails as affected by various levels of CLNS mixture supplementation.

Items	BW (g)			BWG (g)		
	1 wk	3 wk	6 wk	1–3 wk	3–6 wk	1–6 wk
CLNS Mixture (mL/kg Diet)						
0.00	22.86	92.54	191.62 ^a^	4.97	7.08 ^a^	6.03 ^a^
1.50	22.71	90.10	185.02 ^b^	4.81	6.78 ^b^	5.79 ^b^
3.00	22.66	92.42	193.61 ^a^	4.98	7.22 ^a^	6.11 ^a^
SEM	0.06	1.61	4.65	0.11	0.32	0.17
*p* values	0.118	0.963	0.006	0.962	0.007	0.007

CLNS: a mixture of clove and *Nigella sativa* oils; BW: body weight, BWG: body weight gain. Means in the same column with no superscript letters after them or with a common superscript letter following them are not significantly different (*p <* 0.05). *n* = 5.

**Table 3:** Feed intake and feed conversion ratio of Japanese quails as affected by various levels of CLNS mixture supplementation.

Items	FI (g/Day)			FCR (g Feed/g Gain)		
	1–3 wk	3–6 wk	1–6 wk	1–3 wk	3–6 wk	1–6 wk
CLNS Mixture (mL/kg Diet)						
0.00 s	12.47 ^b^	20.30	16.38 ^b^	2.41	2.76	2.58
1.50	12.69 ^b^	21.59	17.14 ^a^	2.54	3.17	2.86
3.00	13.60 ^a^	21.55	17.57 ^a^	2.63	2.86	2.75
SEM	0.10	1.77	0.23	0.06	0.18	0.09
*p* value	0.003	0.052	0.000	0.827	0.116	0.119

CLNS: a mixture of clove and *Nigella sativa* oils; FI: feed intake, FCR: feed conversion ratio. Means in the same column with no superscript letters after them or with a common superscript letter following them are not significantly different (*p <* 0.05). *n* = 5.

**Table 4:** Carcass characteristics of Japanese quails as affected by various levels of CLNS mixture supplementation.

Items	Carcass Traits (% of Slaughter Weight)				
	Dressing	Giblets	Carcass	Breast	Thigh
CLNS Mixture (mL/kg Diet)					
0.00	70.45 ^b^	5.48	64.97 ^c^	43.06 ^b^	22.13 ^b^
1.50	73.35 ^a^	5.80	67.55 ^b^	44.66 ^a^	23.01 ^a^
3.00	73.74 ^a^	5.30	68.45 ^a^	44.78 ^a^	23.90 ^a^
SEM	0.80	0.10	0.75	0.35	0.38
*p* value	0.01	0.231	>0.01	>0.01	>0.01

CLNS: a mixture of clove and *Nigella sativa* oils. Means in the same column with no superscript letters after them or with a common superscript letter following them are not significantly different (*p <* 0.05). *n* = 6.

**Table 5:** The potential role of CLNS mixture supplementation on some liver serum parameters, total protein, and total globulins in Japanese quails.

Items	ALT (U/L)	AST (U/L)	Total Bilirubin (mg/dL)	Total Protein (g/dL)	Albumin (g/dL)	Total Globulins (g/dL)
CLNS Mixture (mL/kg Diet)						
0.00	22.77	285.60	0.65	2.33 ^c^	1.82	0.51 ^b^
1.50	19.58	261.43	0.87	2.96 ^b^	1.83	1.13 ^a^
3.00	16.52	223.13	0.79	3.53 ^a^	1.92	1.61 ^a^
SEM	2.30	19.56	0.06	0.27	0.10	0.21
*p* value	0.124	0.389	0.250	0.016	0.082	0.006

CLNS: a mixture of clove and *Nigella sativa* oils, AST: aspartate aminotransferase, ALT: alanine aminotransferase. Means in the same column with a different superscript letter following them are significantly different (*p <* 0.05). *n* = 6.

**Table 6:** The potential role of CLNS mixture supplementation on blood urea nitrogen (BUN), creatinine, and lipid profile in Japanese quails.

Items	BUN (mg/dL)	Creatinine (mg/dL)	Total Cholesterol (mg/dL)	Triacylglycerol (mg/dL)	HDL-c (mg/dL)	LDL-c (mg/dL)	FFA (mg/dL)
CLNS Mixture (mL/kg Diet)							
0.00	18.85 ^a^	0.79 ^a^	307.60 ^a^	309.39 ^a^	79.24 ^c^	92.49 ^a^	7.23 ^a^
1.50	14.03 ^b^	0.60 ^b^	198.33 ^b^	240.86 ^b^	103.80 ^b^	64.81 ^b^	5.83 ^b^
3.00	11.62 ^c^	0.49 ^c^	171.70 ^c^	181.68 ^c^	123.12 ^a^	51.39 ^c^	4.53 ^c^
SEM	0.50	0.04	20.26	23.81	20.01	8.07	0.39
*p* value	0.022	0.003	0.003	0.001	0.003	0.000	0.000

CLNS: a mixture of clove and *Nigella sativa* oils, BUN: blood urea nitrogen, HDL-c: high-density lipoprotein cholesterol, LDL-c: low-density lipoprotein cholesterol, FFA: free fatty acids. Means in the same column with a different superscript letter following them are significantly different (*p <* 0.05). *n* = 6.

**Table 7:** The potential role of CLNS mixture on antioxidant status in liver homogenates of experimental groups.

Items	MDA (μmol/g Tissue)	GSH (mg/g Tissue)	CAT (μmol H_2_O_2_ Decomposed/g Tissue)	SOD (U/g Tissue)	GPx (μmol NADPH/g Tissue	GR (U/g Tissue)	GST (U/g Tissue)
CLNS Mixture (mL/kg Diet)							
0.00	143.98 ^a^	14.32 ^a^	14.70 ^c^	7.61 ^c^	8.42 ^c^	0.75 ^c^	0.18 ^c^
1.50	102.64 ^b^	30.11 ^b^	23.40 ^b^	20.58 ^b^	12.37 ^b^	1.79 ^b^	0.36 ^b^
3.00	80.44 ^c^	37.36 ^c^	28.94 ^a^	26.60 ^a^	14.91 ^a^	2.26 ^a^	0.57 ^a^
SEM	14.35	4.71	2.21	4.38	0.92	0.34	0.05
*p* value	0.001	0.001	0.001	0.001	0.001	0.001	0.001

CLNS: a mixture of clove and *Nigella sativa* oils, MDA: malondialdehyde, GSH: reduced glutathione, CAT: catalase, SOD: superoxide dismutase, GPx: glutathione peroxidase, GR: glutathione reductase, GST: glutathione S transferase. Means in the same column with a different superscript letter following them are significantly different (*p <* 0.05). *n* = 6.

## Data Availability

Not applicable.
